# Nanotechnology and Nanotoxicology in Retinopathy

**DOI:** 10.3390/ijms12118288

**Published:** 2011-11-23

**Authors:** Dong Hyun Jo, Tae Geol Lee, Jeong Hun Kim

**Affiliations:** 1 Department of Ophthalmology, College of Medicine, Seoul National University, Seoul 110-744, Korea; E-Mail: zodong82@gmail.com; 2 Fight against Angiogenesis-Related Blindness (FARB) Laboratory, Clinical Research Institute, Seoul National University Hospital, Seoul 110-744, Korea; 3 Center for Nano-Bio Technology, Division of Convergence Technology, Korea Research Institute of Standards and Science (KRISS), Daejon 305-340, Korea; E-Mail: tglee@kriss.re.kr

**Keywords:** blood-retinal barrier, diabetic retinopathy, macular degeneration, nanoparticles, retinal degeneration, retinal diseases, toxicology, uveitis

## Abstract

Nanoparticles are nanometer-scaled particles, and can be utilized in the form of nanocapsules, nanoconjugates, or nanoparticles themselves for the treatment of retinopathy, including angiogensis-related blindness, retinal degeneration, and uveitis. They are thought to improve the bioavailability in the retina and the permeability of therapeutic molecules across the barriers of the eye, such as the cornea, conjunctiva, and especially, blood-retinal barriers (BRBs). However, consisting of multiple neuronal cells, the retina can be the target of neuronal toxicity of nanoparticles, in common with the central and peripheral nervous system. Furthermore, the ability of nanoparticles to pass through the BRBs might increase the possibility of toxicity, simultaneously promoting distribution in the retinal layers. In this regard, we discussed nanotechnology and nanotoxicology in the treatment of retinopathy.

## 1. Introduction

Nanoparticles (NPs) are small-sized particles that are nanoscale in three dimensions (http://www.ncbi.nlm.nih.gov/mesh/68053758). In the field of ophthalmology, NPs received attention as one of the novel drug delivery systems (DDS) that overcome the barriers of the eye including cornea, conjunctiva, and blood-retinal barriers (BRB) [1,2]. In particular, retinopathy might be the main target of NP-based medicine in ophthalmology, because it is hard for specific drugs to reach the retina with the appropriate concentration due to restricted permeability caused by BRBs [1–3]. BRB functions as a selective barrier between nervous and circulatory systems [4,5], and consists of inner and outer BRBs. Inner BRB is the dynamic structure consisting of retinal endothelial cells, pericytes, and astrocytes, and outer BRB is the tight junction between retinal pigment epithelium (RPE) cells [5,6].

We previously reported that gold NPs (AuNPs) of which sizes were 20 nm could pass through the BRB, whereas 100 nm GNPs were not observed in any of the retinal layers when they were administered intravenously [7]. These findings suggested that NPs with a small enough diameter might overcome limited penetration of therapeutic agents with larger sizes across the inner BRBs. Similarly, the periocular route is also a promising path for therapeutic NPs, because the sclera has a large surface area and relatively high permeability, so long as NPs could penetrate into the outer BRBs [3].

In this review, we discussed technological advances and toxicology issues of nanoparticles in the treatment of retinopathy.

## 2. Retinopathy as the Target of NP-Based Medicine

**Figure 1:**
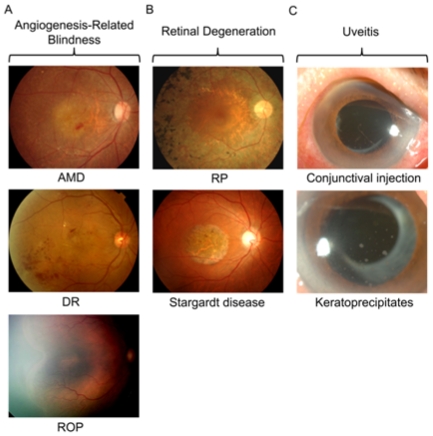
Clinical manifestations of representative diseases of retinopathy, as targets of nanoparticle-based medicine. (**a**) Angiogenesis-related blindness. Subretinal hemorrhage is observed in a patient with age-related macular degeneration (AMD), and retinal neovascularization is a characteristic found in patients with diabetic retinopathy (DR) and retinopathy of prematurity (ROP); (**b**) Retinal degeneration. Retinitis pigmentosa (RP) is characterized by diffusely degenerative retina with pigmented lesions. Stargardt disease is an inherited macular degeneration, and yellowish-white flecks are identified in the macula in this patient; (**c**) In the eye with uveitis, conjunctival injection (top) and keratoprecipitates (bottom) can be observed.

Current therapeutic attempts using various NPs in the treatment of retinopathy are summarized in Table 1, and clinical manifestations of representative diseases with retinopathy are presented in Figure 1.

### 2.1. Angiogenesis-Related Blindness

Angiogenesis-related blindness (ARB) indicates the spectrum of retinal disorders of which pathogenesis is related with pathologic angiogenesis, including age-related macular degeneration (AMD), diabetic retinopathy (DR), and retinopathy of prematurity (ROP) [4]. These three diseases affect mostly elderly, middle-aged, and newborn patients, respectively, resulting in visual deterioration in corresponding age groups. Conventional treatment options for ARB were surgery and focal treatment, such as laser photocoagulation and cryotherapy. In recent years, intravitreal injection of anti-vascular endothelial growth factor (VEGF) monoclonal antibodies (mAb) has been widely performed to treat pathologic angiogenesis. However, despite the considerable effect of anti-VEGF mAb, it has limitations in that repeated injections are inevitable due to relatively short durability, and repeated injections up to seven or eight times a year increased injection-related complications [1,2]. Also, direct inhibition by anti-VEGF mAb is known to influence retinal neuronal cells, aggravate retinal ischemia, and induce mitochondrial disruption of photoreceptor cells [27–29].

NPs are thought to overcome the limitations of current intraocular injection of anti-VEGF mAb, because they can improve therapeutic efficiency by increasing bioavailability [1]. We previously reported that AuNPs inhibit retinal neovascularization via suppression of VEGF receptor-2 activation [8], and Kalishwaralal *et al*. also suggested inhibitory effect of AuNP on VEGF-induced angiogenesis and vascular permeability [9]. The exact mechanisms of antiangiogenic effect of AuNP are yet to be found, however, the binding capacity of AuNP to heparin-binding proteins of certain VEGF isoforms such as VEGF-165 was considered to be one of the possible mechanisms [30]. Interestingly, silicate NPs also evidenced to have an antiangiogenic effect on retinal neovascularization, suggesting shape, mass, or size as well as backbone materials of NP might be important factors in antiangiogenic effect of NPs [15]. Furthermore, silver NPs blocked VEGF-induced proliferation and migration of endothelial cells, and the mechanism might induce apoptosis [16,17]. Another example of NPs exerting antiangiogenic effect is nanoceria, cerium oxide NPs, of which injection resulted in reduction of reactive oxygen species (ROS) and regression of pathologic vascular lesions in Vldlr knockout mice [10].

As previously mentioned, NPs are also utilized as the novel DDS to deliver therapeutic agents to target organs or tissues. For this purpose, polymers including polylactic acid (PLA) and polylactic-co-glycolic acid (PLGA) are widely used [1,31,32]. In the treatment of ARB, nanocapsules encapsulating integrin antagonist peptide or plasminogen kringle 5 (K5) demonstrated to inhibit choroidal neovascularization (CNV) in the laser-induced CNV mice models [11,13]. PLGA nanocapsules encapsulating K5 also ameliorates retinal neovascularization in the oxygen-induced retinopathy (OIR) mice model and vascular leakage in the streptozocin-induced diabetes model [14]. These studies showed that nanocapsules of which sizes were about 300 nm effectively deliver antiangiogenic molecules to the retina and even subretinal spaces where CNV occurred.

Singh *et al*. reported nanocapsules loaded with plasmid of anti-VEGF intraceptor could be conjugated with surface peptide and trasferrin [12]. These NPs are examples of nanoconjugates, engineered NPs harboring bound modules that target specific tissues and cells (http://www.ncbi.nlm.nih.gov/mesh/68058726). PLGA nanocapsules with Flt23K plasmid also allowed targeted gene delivery with intravenous administration, and inhibited progression of pathologic angiogenesis in the laser-induced CNV rat model [12].

### 2.2. Retinal Degeneration

Retinal degeneration, occurred in inherited or genetic ocular diseases such as retinitis pigmentosa (RP) and Stargardt disease, is also one of the targets of NP-based therapeutics in retinopathy. RP is a retinal degenerative disease, initially affecting the rod photoreceptor cells [33]. Stargardt disease is named after Karl Stargardt, who identified and described clinical features patients from two families [34]. From the patients with both diseases, various genetic aberrations were reported, and some of them have been the targets of gene therapy [35]. However, leaving the use of antioxidant aside, there is no definite cure for retinal degeneration, and we have much to do for stable delivery and application of gene therapy [33,35].

Interestingly, nanoceria prevented light-induced degeneration of photoreceptor cells by reducing levels of toxic ROS in the retinal cells [20]. A recent report from the same group demonstrated that nanoceria could extend photoreceptor cell life span in *tubby* mice, which exhibited progressive cochlear and retinal degeneration, resulting in suppression of progression of degenerative changes in the retina when they are injected systemically [21]. These studies suggested the possible action of small NPs mimicking enzymes such as superoxide dismutase and catalase.

Solid lipid nanocapsules loaded with an inhibitor for ceramide signaling pathway lowered retinal ceramide levels and prevented photoreceptors from apoptotic death [19]. The more interesting part of this study was that NPs were administered topically, and therapeutic agents passed through corneal barrier, exerting actions in the retinal layers. Intravitreal injection of gelatin nanoconjugates with basic fibroblast growth factor also prevented photoreceptor degeneration by inhibiting apoptosis in a well-known animal model of retinal degeneration, the Royal College of Surgeon rat [18]. NPs developed by Read *et al*. demonstrated the features of both nanoconjugates and nanocapsules [22]. The poly(ethylene glycol) (PEG) NPs were conjugated with the peptide for ocular delivery (nanoconjugates), and contained an expression cassette for glial cell line-derived neurotrophic factor (nanocapsules). Furthermore, after subretinal delivery of compacted DNA into PEG NPs, gene expression was sustained for up to four months, and degenerative changes were retarded in the animal model of retinal degeneration [23,24].

### 2.3. Uveitis

Uveitis is the inflammation of uveal tissues: the iris, ciliary body, and choroid. In some patients with uveitis, we could observe inflammatory cells in the anterior chamber and corneal precipitates by the slitlamp examination (Figure 1C). Like other organs, to prevent damage related with chronic inflammation, corticosteroid and other immunosuppressive therapies have been utilized in the treatment of uveitis [36]. Unfortunately, subconjunctival or intravitreal injection of corticosteroid has relatively short action, and uveitis itself has a characteristic of frequent relapse. Therefore, biodegradable and non-biodegradable implants of corticosteroid were developed to reduce the injection numbers and enable stable release of therapeutic agents [3,37,38].

NPs also can be an answer for the treatment of uveitis with increased bioavailability and prolonged action. PLA NPs encapsulating betamethasone phosphate reduced clinical response and immunologic reaction in experimental autoimmune uveoretinitis (EAU) rats [26]. PEG-coated NPs with tamoxifen also showed inhibitory effect on inflammation in EAU rats [25]. Interestingly, in this study, intravitreal injection of free tamoxifen did not alter the course of EAU, and after the injection of PEG-coated NPs, NPs were distributed inside and outside of resident ocular cells, suggesting increased biodistribution and effectiveness of therapeutic agents with the aid of NPs.

## 3. Neuronal Toxicity of NPs in Retinopathy

Therapeutic application of NPs in retinopathy has received much attention, however, toxicity on neuronal cells and other resident cells in the retina should be validated before applying NPs to real patients. The retina is a layered structure consisting of three layers of nucleated cells and two plexiform layers [4]. Upon stimulation by light, responses by cone and rod photoreceptor cells are converted to electrical signals and transmitted to the optic nerve via synapses between neuronal cells and bipolar, horizontal, and amacrine cells [39]. If cells participating in this process are damaged or affected by external factors such as therapeutic agents or infectious organisms, visual response may deteriorate. Thus, candidate NPs should be validated not to have definite toxic effect on the retina in *in vitro* and *in vivo* assays before clinical application.

### 3.1. Studies on Nanotoxicology in Retinopathy

In retinopathy, most studies on nanotoxicology have been performed as a part of studies on the therapeutic effect of NPs, and reports focusing solely on the toxic effect of NPs are limited. However, previously mentioned studies on application of NPs in retinopathy mostly have confirmed no evidence of definite toxicity on the retina at the therapeutic dosage by histologic examination, and sometimes electroretinography, which measured electrical response of the retina.

Bourges *et al*. reported the distribution of PLA NPs within the intraocular tissues and their potential to release encapsulated material [40]. The administered concentration was 2.2 mg/mL, and the intensity of inflammation in the retinal layers was increased 6 to 24 hours, however, decreased markedly by 48 hours. Interestingly, NPs were remnant within the RPE cells four months after the intravitreal injection without definite histologic change in the retinal layers. This study demonstrated that PLA NPs, widely used nanocapsules, might be utilized with expectation of sustained delivery of therapeutic agents. Merodio *et al*. also reported prolonged residence of NPs in the vitreous cavity two weeks after a single intravitreal injection [41]. Toxicity was examined with the histologic evaluation and immunohistochemisty of ocular autoantigens, arrestin and rhodopsin. Bovine serum albumin NPs, of about 300 nm and a concentration was 200 mg/mL, did neither induce inflammatory reaction in the retinal tissue nor alter the organization of the retinal layers and expression of retinal autoantigens.

There have been studies evaluating the toxicity according to different types, concentrations, and the polymer tail lengths of NPs [42–45]. In a rabbit model, intravitreal AuNPs at concentrations of 67 μmol/0.1 mL and 670 μmol/0.1 mL did not induce retinal or optic nerve toxicity after 1 week and 1 month, examined by histologic examination via the light microscope [42]. As for PEG-compacted DNA NPs utilized for the treatment of retinal degeneration, subretinal injection of NPs at concentrations of 0.3, 1.0, and 3.0 μg/μL did neither induce infiltration of polymorphonuclear neutrophils or lymphocytes nor increase the markers for inflammatory cells [43]. Hafeli *et al*. reported that iron magnetic NPs coated with the shortest polyehylenoxide tails were more toxic than NPs with longer tails [44]. Interestingly, alteration of surface characteristics of NPs resulted in decreased toxicity on endothelial and RPE cells. Prow *et al*. demonstrated safety and effectiveness of magnetic NPs by comparison with chitosan and poly{[(cholesteryl oxocarbonylamido ethyl) methyl bis(ethylene) ammonium iodide] ethyl phosphate} NPs [45]. In this study, the authors evaluated eyes grossly for RPE abnormalities, retinal degeneration, and inflammation, and concluded that intravitreal and subretinal injection of magnetic NPs were nontoxic to the retina.

### 3.2. Possible Mechanisms of Neuronal Toxicity Induced by NPs

Possible mechanisms of neuronal toxicity of NPs were postulated by various studies. ROS is one of the most widely suggested mechanisms of neurotoxicity of NPs [46–49]. Titanium dioxide (TiO_2_) NPs induced intracellular formation of ROS in neuronal and glial cells, resulting in disturbance of the electrical activity of neuronal networks [46,47]. In a gene expression study regarding oxidative stress and antioxidant, silver NPs caused alteration of gene expression pattern in the tissues from the caudate nucleus, frontal cortex, and hippocampus of mice [48,49]. Manganese and copper NPs induced differential expression of genes regarding dopamine synthesis and metabolism [49]. These data suggested that enzymatic alterations might affect toxicity in dopamine-producing neuronal cells. Besides neuronal cells, microglial cells were reported to be cells most likely to respond to NPs [50,51]. When treated with silica NPs, microglial cells took up NPs as they engulf foreign materials in phagocytosis, increasing the production of intracellular ROS and reactive nitrogen species [50]. In addition, silicate NPs altered cytokine release of microglial cells, suggesting the possibility of affecting surrounding neuronal cells. AuNPs also demonstrated to up-regulate toll-like receptor 2, interlukin 1-alpha, granulocyte macrophage colony-stimulating factor and nitric oxide in microglial cells [51].

Increase in intracellular calcium was observed in the well-differentiated neuronal cell line when they were treated with silica NPs at a concentration that was toxic [52]. These results showed that the effect of NPs on neuronal cell survival might be related to the degree of perturbation of calcium homeostasis. In primary murine cortical networks on neurochips, carbon black, iron, titanium dioxide NPs induced changes of the electrical activity [46]. Liu *et al*. reported that the change of action potential properties was related with depression of voltage-gated sodium current by silver NPs [53]. In hippocampal neurons, silver NPs of 10 μg/mL decreased peak amplitude and overshoot of the evoked single action potential.

### 3.3. Factors Affecting Neuronal Toxicity of NPs

Among the factors that are likely to affect neuronal toxicity of NPs, size and concentration are thought to be the most important ones. In the studies on microglial reponse, 200 nm silica NPs did not show any toxic effect even at relatively high concentrations (292 μg/mL), whereas 50 nm-sized NPs decreased neuronal survival in a dose-dependent way [52]. As for copper NPs, 40 nm-sized NPs exerted the maximum toxic effect when compared with 60 and 80 nm-sized NPs [54]. These data might result from the fact that smaller NPs have more surface molecules per particle available, thereby interacting with surrounding cells or proteins more efficiently [55]. Furthermore, smaller AuNPs (10 nm) demonstrated to be widely distributed in various organ systems including blood, liver, spleen, kidney, testis, thymus, heart, and brain, whereas larger NPs (50, 100, and 250 nm) were detected only in blood, liver, spleen when they were injected intravenously in rats [56]. We also showed that intravenously administered 20 nm-sized AuNPs were observed in all retinal layers including neuronal, endothelial, and peri-endothelial glial cells, however 100 nm-sized AuNPs were not detected in the retina [7]. In this regard, smaller NPs have higher surface molecules and improved penetration into target organs, simultaneously increasing the possibility of toxicity in neuronal cells. However, using 10, 30, 60, and 200 nm sized zinc NPs, Deng *et al*. demonstrated that Zinc NPs manifested no size-dependent toxic effect on neuronal stem cells, only evidencing dose-dependent toxicity [57]. Dose and concentration effect was definitely observed in various studies [46,52–54,57]. As with therapeutic agents with larger scales, NPs should be administered at a proper concentration to induce aimed effects, not toxic effects. Furthermore, although no definite subacute physiological damage was found, the accumulation of AuNPs in the brain and other organs was increased depending on the doses administered in mice [58].

Time might be another important factor in neuronal toxicity. To demonstrate time-dependent dislocation and potential damage of titanium dioxide NPs on central nervous system, female mice were intranasally instilled with NPs every other day. As expected, the group with 30 day-exposure demonstrated significantly increased oxidative damage expressed as lipid peroxidation [59]. Surface charge of NPs also affected toxicity and integrity of the blood-neural barrier [60,61]. In cellular viability, hemolysis, and bacterial viability assays, anionic AuNPs demonstrated less toxicity than cationic ones [60]. Furthermore, in the evaluation of effect of neutral, anionic, and cationic NPs on blood-brain barrier (BBB), neutral and low concentrations of anionic NPs had no effect on BBB integrity, however, cationic NPs and high concentrations of anionic NPs exerted alteration in BBB [61]. In this regard, authors suggested neutral or low concentrations of anionic NPs could be utilized as DDS for therapeutic agents targeted to brain.

There was also a concern that materials constituted of NPs had an influence on neuronal toxicity [46,57]. Gramowski *et al*. reported that titanium dioxide NPs caused oxidation damage in neuronal and glial cells, however, exposure to carbon black and iron NPs at relatively higher concentrations did not induce significant formation of intracellular ROS [46]. Zinc oxide NPs demonstrated only dose-dependent, but no size-dependent toxic effects on neural stem cells [57]. These results might support the concept that zinc or zinc ion itself induced the toxicity, because toxic effects were observed regardless of size of NPs.

**Figure 2:**
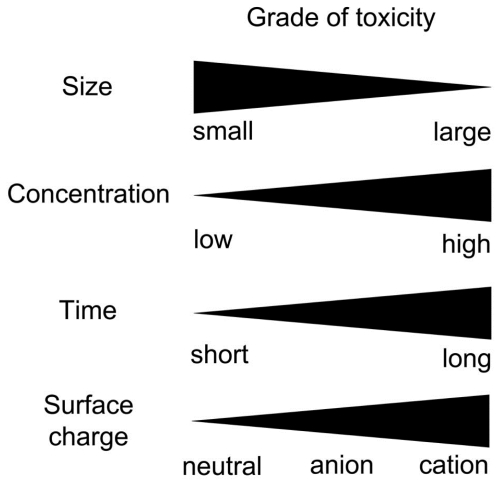
Characteristics of NPs affecting neuronal toxicity.

Factors affecting toxicity of NPs on neuronal cells are summarized in Figure 2.

## 4. NPs on Blood-Retinal Barrier: Increasing the Bioavailability *versus* Affecting the BRB

We previously reported the ability of 20 nm sized AuNPs to pass through BRB [7], and NPs of similar sizes were known to cross BBB [44]. These characteristics may help to increase the bioavailability of therapeutic agents for diseases of the retina and the brain. However, at the same time, increased biodistribution in the retina and the brain can cause more toxicity. Therefore, researchers and clinicians who want to use NPs as therapeutic agents should carefully determine the size and concentration of NPs to effectively increase bioavailability and minimize possible toxicities.

Although the inhibitory effect of AuNPs on vascular permeability in retinal endothelial cells might be induced by suppression of VEGF receptor or direct inhibition of specific isoforms of VEGF, this effect demonstrated the possible action of NPs on the restoration of diseased BRB [9]. On the contrary, there was a report showing the disturbance of the integrity of BBB caused by emulsifying wax NPs [61]. NPs of small sizes could pass through the blood neural barriers, but the effect of NPs on the barriers might be different according to the types or concentrations of NPs. In this regard, the possibility of unwanted effects on BRB should be considered in the development of therapeutic modalities using NPs.

## 5. Nanotechnology and Nanotoxicology in Retinopathy: Friends or Foes?

In general, there has been a growing concern that nanotechnology outgrows nanotoxicology in neuronal diseases. AuNPs and silicate NPs are known to be less toxic than NPs of other materials, however, the exact toxicity profiles of those NPs are yet to be presented, and there is also a possibility that neuronal toxicities of NPs can be different according to small differences in the production procedure or characteristics of NPs, even though the backbone materials are the same. As previously mentioned, size, dose, dosing time, surface charge, and other factors can attribute differences in neuronal toxicities of NPs.

Magnetic NPs could be protected from acidic erosion when coated with amorphous silica, stabilizing the NPs and reducing toxicities in *in vitro* cellular toxicity assays [62]. According to the charges of side chains, the same NPs exerted different toxicities in various cell types [60]. Smaller NPs could penetrate the various barriers including BBB and BRB [45,53], and with these NPs, we could administer lesser concentrations to the patients. With current technology regarding production of NPs, we can change these characteristics of NPs, minimizing neuronal toxicities.

Some authors suggested plausible recommendations for studies on NPs with regard to concerns on nanotoxicology [55]. The properties of NPs should be characterized sufficiently before studies with those NPs are performed. The parameters include chemical composition, size (with distribution), surface characteristics, presence of coating, degree of aggregation, water solubility, and zeta potential. These guidelines support the notion that certain characteristics of NPs might influence toxicities, and consideration of those properties might improve methods to lessen toxicity and activate discussion about possible modification to improve toxicity profiles of NPs. Progressive nanotechnology brought about the issue of nanotoxicology, however, the answer for reducing toxicity of NPs will be from advances in nanotechnology.

## 6. Future Directions

In this review, we discussed presumptive applications and possible toxicities of NPs in retinopathy. Conventional treatment options for retinopathy, include surgery, focal treatment, and intravitreal anti-VEGF mAb, and have considerable therapeutic effects on patients with ARB, retinal degeneration, and uveitis. However, NPs still have possibilities for wide application in retinopathy, because NPs can pass through BRB or other barriers in the eye, and prolonged residence of NPs in the eye can reduce treatment numbers, which is one of the most bothersome problems of intravitreal anti-VEGF mAb. In considering characteristics of NPs that affect neuronal toxicities and BRB, researchers should focus on the development of NPs with enhanced efficacy and minimal toxicity. Current technology regarding the production of NPs can help increase efficacy with modification of surface charge or conjugated molecules, and cautiousness concerning possible toxicities of NPs on the retina and even the nervous system, will reduce toxicities. As nanotechnology progresses, we look forward to the development of “safe, but effective” NP technology in retinopathy, which will help patients who have suffered from catastrophic visual loss.

## Figures and Tables

**Table 1:** Retinopathy as the target of nanoparticle-based therapeutic approach.

Material	Type	Size (nm)	Concentration	Animal model	Administration	Reference
***Angiogenesis-related blindness***						
Gold	Nanoparticle	20	1 μM	OIR	IVT	[8]
Gold	Nanoparticle	50	500 nM	Cell work only	N/A	[9]
Nanoceria	Nanoparticle	3–5	1 mM	VLDLR KO	IVT	[10]
PLA/PLA-PEO	Nanocapsule	302	0.12 mg/μL	Laser CNV	IVT	[11]
PLGA	Nanoconjugate	270–420	N/A	Laser CNV	IV	[12]
PLGA-chitosan	Nanocapsule	260	N/A	Laser CNV	IVT	[13]
PLGA-chitosan	Nanocapsule	260	N/A	OIR	IVT	[14]
Silicate	Nanoparticle	57	10 μg/mL	OIR	IVT	[15]
Silver	Nanoparticles	50	500 nM	Cell work only	N/A	[16,17]
***Retinal degeneration***						
Gelatin	Nanoconjugate	585	N/A	RCS rat	IVT	[18]
Lipid	Nanocapsule	40–200	N/A	rd10 mouse	Topical	[19]
Nanoceria	Nanoparticle	−5	0.1–1 μM	Light-induced RD	IVT	[20]
Nanoceria	Nanoparticle	N/A	1 mM	*tubby* mouse	Intracardial	[21]
PEG	Nanoconjugate	175.9	N/A	Light-induced RD	Subretinal	[22]
PEG	Nanocapsule	−8	3.06 μg/μL	rds^+/−^ mouse	Subretinal	[23,24]
***Uveitis***						
PEG	Nanocapsule	95–112	N/A	EAU	IVT	[25]
PLA	Nanocapsule	100–200	N/A	EAU	IV	[26]

CNV: choroidal neovascularization; EAU: experimental autoimmune uveitis; IV: intravenous; IVT: intravitreal; KO: knockout; N/A: not applicable or not available; NP: nanoparticle; OIR: oxygen-induced retinopathy; PEG: poly(ethylene glycol); PEO: poly(ethylene oxide); PLA: polylactic acid; PLGA: polylactic-co-glycolic acid; RCS: Royal College of Surgeon; RD: retinal degeneration; VLDLR: very low density lipoprotein receptor.
